# Does women’s decision-making autonomy matter in utilization of antenatal care services in India? An analysis from nationally representative survey

**DOI:** 10.1371/journal.pone.0308576

**Published:** 2024-08-22

**Authors:** Koyel Majumder, Mithun Sarkar, Rahul Mallick, Sabbir Mondal, Pradip Chouhan

**Affiliations:** 1 Department of Geography, University of Gour Banga (UGB), Malda, West Bengal, India; 2 Department of Geography, Rammohan College, Kolkata, West Bengal, India; Oxford University: University of Oxford, UNITED KINGDOM OF GREAT BRITAIN AND NORTHERN IRELAND

## Abstract

The primary goal is to examine the association between women’s decision-making autonomy and utilization of antenatal care services among ever-married women in India. The entire study has been accomplished with the help of secondary data composed from the latest round of the National Family Health Survey (NFHS-5) conducted during 2019–21. A total of 34,618 ever-married women aged 15–49 with at least one live birth preceding five years of the survey have been taken into consideration in this study. Bivariate and multivariate analyses were conducted for proper illustration of the outcome. A sizable proportion of women did not fulfill the WHO-recommended criterion of obtaining ANC services. Utilization of full ANC services is found in some regions of southern, eastern, northern, and northeastern states, and in some districts of Gujarat. After adjusting the other explanatory variables, the result of multivariate analysis indicates that women’s autonomy is significantly and positively associated with the utilization of full antenatal care services. Women who participate actively in decision-making are more likely to use full ANC services (AOR: 1.316, 95% CI: 1.197–1.446, p<0.05). Additionally, likelihood of utilization of full ANC services is high among women aged 25–34 years, are educated, have access to the media, come from richest wealth quintile, and are from southern, western, and eastern regions. Therefore, appropriate measures should be adopted to eliminate gender bias and promote women’s empowerment for the overall improvement of maternal health as well as societal health.

## 1. Introduction

Maternal mortality is a pressing issue all over the world nowadays, especially in underdeveloped and developing countries. As per the recent World Health Organization (WHO) report, every two minutes one maternal death occurred, almost 800 women died every day and about 287,000 women died in 2020 due to pregnancy-related complications and childbirth [1]. Approximately 95% of these deaths occurred in developing and under-developed countries [1]. But most of these deaths can be prevented through proper utilization of maternal health care services [2, 3] which in turn can support for the betterment of women’s health. Improving maternal health is one of the goals of both Millennium Development Goals (MDGs) and Sustainable Development Goals (SDGs) in addition to targeting the reduction of maternal mortality rate by three quarters between 1990 to 2015 [4, 5] and less than 70 deaths per 100,000 live births by the end of 2030 respectively [6]. Globally, Maternal Mortality Rate (MMR) has declined from an estimated 385 per 100,000 live births in 1990 to 216 in 2015, reduced by 44% over the last 25 years [7]. In India, an estimated MMR was 130 per 100,000 live births in 2014–16 and it has declined to 122 maternal deaths in 2015–17 and 113 in 2016–18. There are large variations in MMR ranging across the states, 215 per 100,000 live births in Assam and 43 per 100,000 live births in Kerala [8].

Former studies have reported that improper utilization of maternal health care services leads to an increase in both high maternal morbidity and mortality [9, 10]. Proper utilization of maternal health care services during pregnancy and childbirth is not only crucial for the improvement of maternal health but also reduces both childhood morbidity and mortality. Maternal health care consists of three basic phases, i.e. antenatal care (during pregnancy), delivery care (during childbirth), and postnatal care (after delivery). Despite all three types of maternal health care having important benefits, antenatal care (ANC) is the most important care [11] because ANC can help women to reduce both maternal morbidity and mortality by providing quality services and encouraging women to deliver at a health facility or deliver with a skilled birth attendant [12]. Antenatal care is a care that is given to a woman from the time of conception to prior delivery by the skilled health provider in order to maintain the health of both mother and fetus [13, 14]. A pregnant woman without any complications should complete a minimum of four antenatal visits, and the first one should take place within the first trimester of pregnancy, should consume at least 100 IFA tablets and should take at least two TT injections [12, 15–17]. Proper ANC can detect the risk factors regarding obstetric complications and help women to educate about the risks during pregnancy [18]. Globally, only 66% of women have accessed WHO recommended four or more ANC services whereas the coverage of four or more ANC is 24% in countries of sub-Saharan Africa and over 90% in many countries including those in Europe, Latin America, and the Caribbean [19].

Women’s autonomy is a very important element for the overall improvement of maternal health as well as societal health. In general, women’s autonomy entails having the capacity and capability for women to freely make decisions regarding their lives. Women’s autonomy is a vast concept and there is no universal accepted definition that represents this multidimensional concept [20]. It indicates control over assets, traditional beliefs or principles, self-confidence, and inner power to overcome external barriers [21]. Basu defined women’s autonomy as the ability and liberty to perform independently like the capability to go to health facilities or markets, to make decisions regarding household purchases or contraceptive use without seeking anyone’s permission [22]. Other scholars explained autonomy as the psychological, technical, and social ability to receive information and to apply it as a base for decision-making regarding one’s personal apprehension and companions [23]. Mason defined women’s autonomy as the capability to take and implement autonomous decisions concerning to individual matters of importance to their lives and families [24]. Generally, autonomy is measured by using three dimensions: right to access and control over finances, capability to make a decision freely, and freedom of mobility [25, 26]. Former studies conducted in Ghana [27], Nigeria [28], Ethiopia [29], Bangladesh [30, 31], Nepal [32], and India [33] have reported that there is a positive relationship between Women’s autonomy and utilization of different types of maternal health care services.

There are numerous socio-demographic, economic and accessibility related factors that influence women in the utilization of antenatal care services in India [34–37] but the lack of women’s autonomy, another crucial determinant that may hinder women from receiving proper ANC services, is often overlooked in the country. In this background of higher occurrence of maternal mortality in India, it is important to highlight the link between women’s decision-making autonomy and utilization of ANC services. It would ameliorate one’s perception regarding the importance of maternal health for the development of a nation and take measures for policymakers to promote the utilization of antenatal care in the country. However, while there have been numerous studies regarding utilization of antenatal care services in India but no studies specifically emphasized on how women’s decision-making autonomy related to utilization of antenatal care services. Previous study [33] examined the association between women’s autonomy and utilization of all three stages of maternal health care in India, namely antenatal care, delivery care, and postnatal care. The current study, on the other hand, attempts to determine the association between women’s decision-making autonomy and utilization of antenatal care services in India. Initially, we have associated women’s decision-making autonomy with the utilization of each four main components of antenatal care services individually. Then we employed the dependent variable ‘full ANC’ which comprises these four components and finally associated with the key predictor variable i.e. women’s decision-making autonomy. Thus, to address this gap, the present study attempts to examine the association between women’s decision-making autonomy and utilization of antenatal care services among ever-married women aged 15–49 years in India using the most current data of the National Family Health Survey, 2019–21.

## 2. Materials and methods

### 2.1 Data source

The analysis is based on the National Family Health Survey (NFHS-5) dataset from the most recent Demographic and Health Survey in India, which was conducted in 2019–2021. The NFHS-5 is a nationally representative large-scale sample survey and gathers information from 636,699 households with a response rate of 98%, 101,839 men (aged 15–54 years) with a response rate of 92% and 724,115 women (aged 15–49 years) with response rate of 97%. The NFHS-5 provides information on household characteristics, level of fertility, infant and child mortality, maternal and child healthcare utilization, maternal and child health, family planning methods, HIV/AIDS, anemia, hypertension and women empowerment. The NFHS-5 provides information up to the district level across all state and union territories in India. The survey used the two-stage stratified cluster sampling technique for selected samples.

### 2.2 Study design and sample size

The study has adopted a cross-sectional design based on the latest dataset of the NFHS-5. Here we used the individual-level KR file of NFHS-5. A total of 724,115 samples were collected from women aged 15–49 years and among them 232,920 are ever-married women who have at least one live birth in the past 5 years before the survey. Here 34,618 women found who have women empowerment data. So, this study analyzed 34,618 ever-married women aged 15–49 years and before the survey who had at least one live birth in the past five years.

### 2.3 Outcome variables

The outcome variable of the present study is ‘full ANC’ which comprises the four main components of antenatal care services (i.e. four or more ANC visits, ANC check up within 1^st^ trimester, taken 100 or more IFA tablets, taken at least two TT injections). A mother considered to have received ‘full ANC’ if she fulfilled all four criteria. Definition of ‘full ANC’ is consistent with the previous studies [15–17]. For empirical analysis, all five variables were divided into binary variables (‘0’ indicates less than four ANC visits, ANC check up after 1^st^ trimester, taken <100 IFA tables, taken <2 TT injection, not received full ANC, and ‘1’ indicates, four or more ANC visits, ANC check up within 1^st^ trimester, taken 100 or more IFA tablets, taken at least two TT injection, received full ANC).

### 2.4 Predictor variables

In this study women’s decision-making autonomy is considered as a key predictor variable and its measure is based on four individual questions asked to the women during the survey of NFHS-5. The four questions were about the person who usually decides (1) on respondent’s health care; (2) on visits to family or relatives; (3) on large household purchases; and (4) what to do with the money the husband earns. Each question had five options, such as respondent alone, respondent and husband/partner, husband/partner alone, someone else and other. The first two responses consider women’s involvement in decision-making and it’s assigned as ‘1’. And other three responses consider no women’s involvement in decision-making autonomy and it is assigned as ‘0’. After that, all four variables are added, the final score ranging from 0 to 4 are then divided the score into three categories. The score ‘4’ indicates women who have high decision-making autonomy, scores ‘2’ and ‘3’ indicate women with moderate decision-making autonomy, and scores ‘0’ and ‘1’ indicate women with low decision-making autonomy. This is consistent with the previous study conducted in India [33].

### 2.5 Confounding variables

The study considers socio-demographic variables as confounding variables i.e. women’s age (15–24, 25–34 and 35–49 years); age at marriage (less than 18 years and more than 18 years); birth order (first, second, third and four or more); place of residence (rural and urban), religion (Hindu, Muslim, Christian and others); Caste (Scheduled Caste, Scheduled Tribe, Other Backward Classes and others); women’s education and her husband’s education level (no education, primary, secondary and higher); mass media exposure (measured by frequently watching TV, listening radio, reading newspaper and magazine. Mass media exposure was further classified into 2 groups where ‘no’ mass media exposure indicates those who do not access these three types of mass media, and ‘yes’ indicates those who accessed at least one of the three types of mass media. Wealth quintile (poorest, poorer, middle, richer, and richest) and for geographical distribution of ANC services, we used the NFHS-5 classification of regions, which divides Indian states and union territories in to six regions (north, central, east, northeast, west and south) [38].

### 2.6 Statistical analysis

Descriptive statistics have been used to show the percentage distribution of study participants by various socio-demographic characteristics and key predictor. We also calculated the percentage distribution of outcome variables (four or more ANC visits, ANC check up within 1^st^ trimester, taken 100 or more IFA tablets, taken at least two TT injections) by key predictor and confounding variables. The sample weight was used to estimate the result. The differences in the utilization of antenatal care by the selected explanatory variables were tested using Pearson’s chi-square statistics. We used the binary logistic regression model to estimate the crude and adjusted association between women’s decision-making autonomy and different components of the utilization of antenatal care. The results of logistic regression are presented in odds ratio with 95% confidence interval (CI). All the statistical analyses have been done by using Stata version 17.0 (StataCorp LLC, College Station, TX, USA).

## 3. Results

Table 1 represents socio-demographic characteristics of 34,618 ever-married women aged 15–49 who had at least one live birth preceding five years of survey in India. Majority of the respondents (62.36%) reported that they had high involvement in household decision-making autonomy, 58.77% women belonged to 25–34 years age group, 34.85% women married before the legal age (<18 years) of marriage, 15.33% women had four birth order or more and 73.61% women resided in rural areas. 78.67% women belonged to Hindu religion and almost half of women (46.77%) were from Other Backward Classes. Only 15.65% women and 16.77% women’s partners completed higher education. 71.40% women exposed to mass media, 24.21% women belonged to poorest wealth quintile and 27.32% women were from central region.

**Table 1:** Socio-demographic characteristics of respondents (N = 34,618).

Variables	Frequency (Percentage)
	(N = 34,618)
**Decision Making Autonomy**	
Low	5,905 (18.32)
Medium	6,501 (19.31)
High	22,212 (62.36)
**Women’s Age (years)**	
15–24	10,705 (33.16)
25–34	20,598 (58.77)
35–49	3,315 (8.08)
**Age at Marriage (years)**	
<18	11,174 (34.85)
≥18	23,163 (65.15)
**Birth order**	
First	8,775 (26.03)
Second	13,373 (39.84)
Third	6,719 (18.80)
Four or more	5,751 (15.33)
**Place of residence**	
Urban	7,074 (26.39)
Rural	27,544 (73.61)
**Religion**	
Hindu	25,251 (78.67)
Muslim	5,118 (16.95)
Christian	2,769 (1.99)
Others	1,480 (2.39)
**Caste**	
Scheduled Caste	7,044 (24.60)
Scheduled Tribe	7,042 (10.32)
Other Backward Classes	13,376 (46.77)
Others	5,360 (18.32)
**Education Level**	
No education	7,518 (20.84)
Primary	4,508 (12.47)
Secondary	17,833 (51.04)
Higher	4,759 (15.65)
**Partner’s Education**	
No education	5,286 (14.91)
Primary	4,527 (13.29)
Secondary	19,352 (55.03)
Higher	5,453 (16.77)
**Mass Media Exposure**	
No	10,139 (28.60)
Yes	24,479 (71.40)
**Wealth Quintile**	
Poorest	9,223 (24.21)
Poorer	8,135 (22.05)
Middle	6,745 (19.67)
Richer	5,926 (18.19)
Richest	4,589 (15.88)
**Region**	
North	6,575 (13.93)
Central	8,794 (27.32)
East	6,722 (26.44)
Northeast	5,080 (3.57)
West	3,083 (11.87)
South	4,364 (16.87)

Significant differences are observed in the utilization of ANC services by the level of decision-making autonomy and socio-demographic characteristics (Table 2). The utilization of four or more ANC visits (64.77%), consumption of 100 or more IFA tablets (65.99%), taking at least two TT injections (65.27%), and full ANC services (67.03%) was high among women with high involvement in decision-making autonomy. The frequency of receiving four or more ANC (60.34%), initiation of first ANC within first trimester of pregnancy (59.82%), consumption of 100 or more IFA tablets (60.64%) and full ANC (61.41%) was high among women aged 25–34 years compare to others. The women who got married after 18 years of age had major tendency of receiving all components of ANC along with full ANC (75.86%) compared to women who were married before the legal age of marriage. Birth order is another important variable that significantly (p = 0.001) influenced on likelihood of receiving full ANC services. Women with higher birth order (7.69%) were less likely to receive full ANC than women with lower birth order. From the residential point of view, women resided in rural areas (70.48%) were more prone to receive full ANC services. The likelihood of receiving four or more ANC services (74.37%), initiation of first ANC within first trimester of pregnancy (74.01%), consumption of 100 or more IFA tablets (75.12%), likelihood of taken at least two TT injections (73.92%) and full ANC services (76.46%) was high among Hindu women compare to others. Among the social group/caste variables, receiving four or more ANC services (40.64%), utilization of first ANC within first trimester (41.38%), consumption of 100 IFA tablets (40.44%), receiving at least two TT injections (41.13%), and utilization of full ANC (40.86%) services was high among those who belong to other backward classes. It is also found that utilization of all the components of ANC services along with full ANC increased with the increasing level of both maternal and paternal education. The percentage of women (57.02%) and their partners (59.05%) who had completed secondary education were more likely to receive all components of full ANC services compared to those who have no formal education. Women who exposed to mass media had higher proportion of receiving full ANC services (84.22%). The proportion of full ANC services also increased with the increasing level of household wealth. Women who belonged to richer household wealth were more active to utilize full ANC services (23.66%). Likelihood of receiving full ANC services was also varied across geographical regions. Utilization of full ANC is found to be high in the southern region (22.84%) and low in the northeast region (11.97%).

**Table 2:** Distribution of antenatal care services by socio-demographic characteristics.

Variables	Four or more ANC visits		Antenatal check up within first trimester		Taken 100 or more IFA tablets		Taken at least two TT injections		Full ANC	
	Percentage (frequency)	*p*- value	Percentage (frequency)	*p—*value	Percentage (frequency)	*p*—value	Percentage (frequency)	*p*—value	Percentage (frequency)	*p*—value
**Decision Making Autonomy**										
Low	15.59 (2364)	0.001	16.30 (3004)	0.752	14.64 (1678)	0.001	16.00 (3448)	0.001	13.44 (821)	0.001
Medium	19.64 (2977)		18.65 (3438)		19.36 (2219)		18.73 (4036)		19.53 (1193)	
High	64.77 (9819)		65.05 (11989)		65.99 (7562)		65.27 (14068)		67.03 (4094)	
**Women’s Age (years)**										
15–24	29.78 (4514)	0.001	30.32 (5588)	0.001	29.24 (3351)	0.001	30.52 (6578)	0.351	28.60 (1747)	0.001
25–34	60.34 (9148)		59.82 (11026)		60.64 (6949)		59.04 (12724)		61.41 (3751)	
35–49	9.88 (1498)		9.86 (1817)		10.11 (1159)		10.44 (2250)		9.99 (610)	
**Age at Marriage (years)**										
<18	27.60 (4163)	0.001	29.23 (5360)	0.001	26.16 (2981)	0.001	30.89 (6625)	0.001	24.14 (1471)	0.001
≥18	72.40 (10921)		70.77 (12976)		73.84 (8416)		69.11 (14821)		75.86 (4623)	
**Birth order**										
First	36.73 (5569)	0.001	35.45 (6534)	0.001	37.63 (4312)	0.001	35.86 (7729)	0.001	41.09 (2510)	0.001
Second	37.64 (5706)		36.59 (6743)		37.67 (4317)		35.30 (7608)		37.69 (2302)	
Third	15.30 (2319)		15.99 (2947)		14.60 (1673)		16.11 (3471)		13.52 (826)	
Four or more	10.33 (1566)		11.97 (2207)		10.10 (1157)		12.73 (2744)		7.69 (470)	
**Place of residence**										
Urban	25.06 (3799)	0.001	23.29 (4293)	0.001	26.67 (3056)	0.001	22.26 (4797)	0.001	29.52 (1803)	0.001
Rural	74.94 (11361)		76.71 (14138)		73.33 (8403)		77.74 (16755)		70.48 (4305)	
**Religion**										
Hindu	74.37 (11274)	0.035	74.01 (13640)	0.003	75.12 (8608)	0.001	73.92 (15931)	0.001	76.46 (4670)	0.023
Muslim	14.58 (2211)		14.84 (2735)		12.84 (1471)		14.75 (3179)		12.82 (783)	
Christian	6.58 (998)		6.74 (1242)		7.28 (834)		6.78 (1461)		6.50 (397)	
Others	4.47 (677)		4.42 (814)		4.76 (546)		4.55 (981)		4.22 (258)	
**Caste**										
Scheduled Caste	20.07 (2876)	0.001	20.46 (3565)	0.001	20.20 (2186)	0.001	21.13 (4300)	0.001	19.20 (1118)	0.001
Scheduled Tribe	19.76 (2831)		19.76 (3443)		20.27 (2194)		20.12 (4095)		19.25 (1121)	
Other Backward Classes	40.64 (5823)		41.38 (7212)		40.44 (4377)		41.13 (7372)		40.86 (2379)	
Others	19.53 (2799)		18.41 (3208)		19.09 (2066)		17.62 (3586)		20.69 (1205)	
**Education Level**										
No education	15.24 (2311)	0.001	17.42 (3210)	0.001	13.58 (1556)	0.001	19.01 (4096)	0.001	11.17 (682)	0.001
Primary	11.24 (1704)		11.72 (2161)		10.55 (1209)		12.04 (2595)		9.04 (552)	
Secondary	55.62 (8432)		53.93 (9940)		55.59 (6370)		53.13 (11450)		57.02 (3483)	
Higher	17.90 (2713)		16.93 (3120)		20.28 (2324)		15.83 (3411)		22.77 (1391)	
**Partner’s Education**										
No education	10.44 (1583)	0.001	12.01 (2213)	0.001	9.81 (1124)	0.001	13.03 (2809)	0.001	7.89 (482)	0.001
Primary	11.14 (1689)		11.48 (2115)		10.98 (1258)		12.13 (2615)		10.09 (616)	
Secondary	58.83 (8919)		57.47 (10592)		58.84 (6743)		57.08 (12301)		59.05 (3607)	
Higher	19.58 (2969)		19.05 (3511)		20.37 (2334)		17.76 (21552)		22.97 (1403)	
**Mass Media Exposure**										
No	20.88 (3165)	0.001	23.89 (4404)	0.001	19.75 (2263)	0.001	26.04 (5613)	0.001	15.78 (964)	0.001
Yes	79.12 (11995)		76.11 (14027)		80.25 (9196)		73.96 (15939)		84.22 (5144)	
**Wealth Quintile**										
Poorest	18.57 (2815)	0.001	21.08 (3886)	0.001	18.12 (2076)	0.001	23.78 (5125)	0.001	14.91 (911)	0.001
Poorer	21.02 (3187)		21.65 (3991)		20.06 (2299)		22.48 (4845)		17.91 (1094)	
Middle	21.43 (3249)		20.35 (3750)		20.87 (2392)		19.83 (4273)		20.97 (1281)	
Richer	20.73 (3142)		19.96 (3678)		21.18 (2427)		18.48 (3983)		23.66 (1445)	
Richest	18.25 (2767)		16.96 (3126)		2265 (1977)		15.43 (3326)		22.54 (1377)	
**Region**										
North	21.66 (3283)	0.001	21.37 (3939)	0.001	20.14 (2308)	0.001	19.63 (4230)	0.001	20.22 (1235)	0.001
Central	20.52 (3111)		22.85 (4211)		19.24 (2205)		24.54 (5289)		15.78 (964)	
East	15.55 (2358)		17.39 (3205)		16.18 (1854)		19.05 (4106)		16.60 (1014)	
Northeast	13.30 (2016)		13.74 (2533)		14.27 (1635)		14.71 (3171)		11.97 (731)	
West	11.71 (1775)		9.85 (1815)		11.12 (1274)		8.71 (1877)		12.59 (769)	
South	17.26 (2617)		14.80 (2728)		19.05 (2183)		13.36 (2879)		22.84 (1395)	

Note: P-value: Significance level of chi-square statistic

Table 3 represents result of unadjusted and adjusted logistic regressions on the components of antenatal care services by level of decision-making autonomy among women aged 15–49 in India. Here it is observed that the odds of receiving all the components of ANC services increases among those women who had medium and high involvement in decision-making autonomy. Odds of receiving four or more ANC visits was high among the women who had medium decision-making autonomy (COR: 1.28, 95% CI: 1.17–1.40) (AOR: 1.19, 95% CI: 1.08–1.31). Besides, women with high decision making autonomy had a higher likelihood of taking 100 or more IFA tablets (AOR: 1.24, 95% CI: 1.14–1.34) and taking at least two TT injections (COR: 1.30, 95% CI: 1.20–1.42) (AOR: 1.33, 95% CI: 1.21–1.45).

**Table 3:** Binary logistic regression models for utilization of the components of antenatal care services in India.

Variables	Received at least four ANC visits		Antenatal checkup within first trimester		Taken 100 or more IFA tablets		Taken at least two TT injections	
	COR (95% CI)	AOR (95% CI)	COR (95% CI)	AOR (95% CI)	COR (95% CI)	AOR (95% CI)	COR (95% CI)	AOR (95% CI)
**Decision-making autonomy**								
Low ^**®**^								
Medium	1.28* (1.17–1.40)	1.19* (1.08–1.31)	1.01 (0.92–1.12)	0.98 (0.88–1.09)	1.27 *(1.16–1.39)	1.19* (1.08–1.31)	1.24* (1.12–1.38)	1.23* (1.10–1.37)
High	1.11* (1.04–1.20)	1.13* (1.04–1.22)	1.03 (0.95–1.11)	1.04 (0.96–1.14)	1.22* (1.14–1.32)	1.24* (1.14–1.34)	1.30* (1.20–1.42)	1.33* (1.21–1.45)

Note: ® Reference category

*p<0.05; COR: Crude odds ratio; AOR: Adjusted odds ratio; CI: Confidence Interval

Table 4 depicts the result of binary logistic regression models for utilization of full antenatal care services in India. It is found that women with medium (AOR: 1.23, 95% CI: 1.10–1.38) and high (AOR: 1.31, 95% CI: 1.19–1.44) decision-making autonomy had a higher likelihood of utilization of full ANC services compared to those women who had no involvement in decision-making autonomy.

**Table 4:** Binary logistic regression models for utilization of full antenatal care services in India.

Variables	Full ANC	
	COR (95% CI)	AOR (95% CI)
**Decision-making autonomy**		
Low ^**®**^		
Medium	1.32* (1.19–1.46)	1.23* (1.10–1.38)
High	1.29* (1.19–1.41)	1.31* (1.19–1.44)

Note: ® Reference category

*p<0.05; COR: Crude odds ratio; AOR: Adjusted odds ratio; CI: Confidence Interval

**Fig 1:**
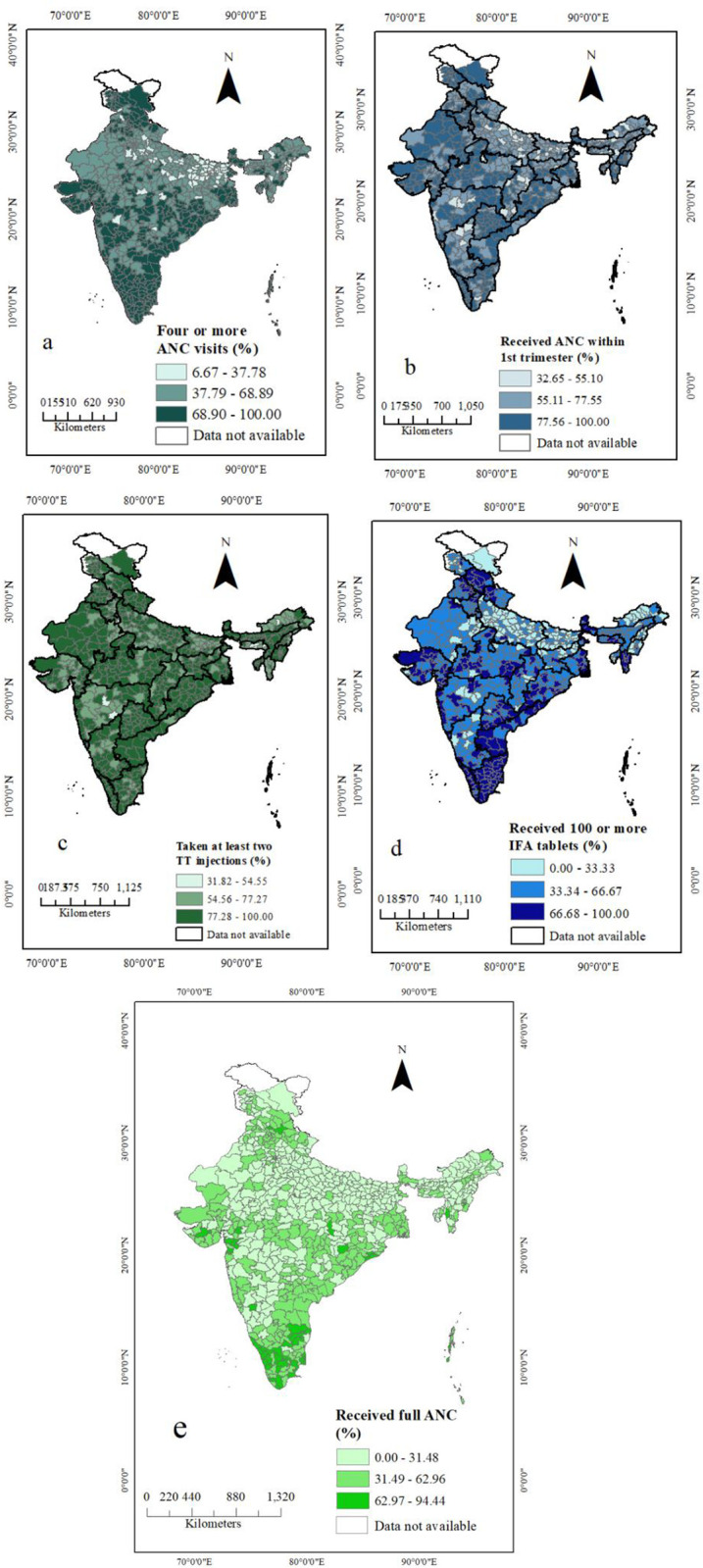
District level patterns of different components of ANC: (a) Four or more ANC visits, (b) received ANC within 1^st^ trimester, (c) received at least two TT injections, (d) received 100 or more IFA tablets, (e) received full ANC.

Fig 1(A)–1(E) illustrates district level patterns of utilization of different components of antenatal care services in India. Utilization of full ANC services which extended from 62.97% to 94.44% is found in some regions of southern, eastern, northern, and northeastern states, and in some districts of Gujarat. A substantial proportion of women did not fulfill the benchmark of WHO recommendations till now and it is observed in almost every part of India especially in northern, northeastern and western parts of India.

## 4. Discussion

The current study intended to investigate the relationship between women’s decision-making autonomy and antenatal care service utilization among ever-married women using the latest round of nationally representative samples. It is found that a great percentage of ever-married women had a high (62.36%) and medium (19.31%) level of decision-making autonomy in India. Women’s autonomy is a very significant indicator in terms of utilization of antenatal care services (ANC) among the women. Result shows that women’s autonomy is significantly and positively associated with the utilization of full antenatal care services after adjusting the other explanatory variables. The findings are in line with prior studies conducted in Ethiopia [20, 39], in Nigeria [28], in Sub-Saharan African countries [40], in Bangladesh [31], in Nepal [32] and in India [33]. The observation supports earlier research findings in several nations that women’s participation in decision-making autonomy leads to higher utilization of antenatal care services [41, 42]. Maitra [43] portraited that Indian women who had a high involvement in household decision-making autonomy were more likely to seek antenatal care services during pregnancy. Similarly, women who handled daily household purchasing decisions were more likely to utilize ANC in India [44]. Another study conducted in a north Indian city [26] explained that women’s autonomy has a greater impact on utilization of ANC services as well as other important explanatory variables like education. The results of these studies indicate that women’s autonomy has a positive impact on the likelihood of enjoying maternal health care services. When women’s autonomy is bounded by prohibiting their movement, participation in decision-making and financial liberty, the outcomes are comparatively lower in the utilization of maternal health care services among the women. Another finding in Ethiopia depicts that women with opposite attitudes towards wife beating had higher odds of likelihood of all three types of maternal health care services. Women with higher decision-making autonomy were more likely to utilize four or more antenatal care services [29].

There are some limitations in the present study. First, due to the cross-sectional design of the study, it is difficult to derive causal relationship between women’s decision-making autonomy and ANC utilization. Second, the study used the data that included the information regarding the utilization of antenatal care services preceding the five years of survey. So, it is impossible to rule out the chance of memory bias. Moreover, women’s decision-making autonomy is calculated based on only four dimensions regarding respondent’s liberty to freely make decisions. But there might be other important aspects of autonomy that could not be included due to limited information. In spite of that, the dimensions of autonomy have been used in this study is significantly associated with utilization of antenatal care services in India. Women’s decision-making autonomy has positively influenced the utilization of ANC services in India. However, antenatal care utilization helps the women by early detection of obstetric complications and provides appropriate treatment and care and thus it act as a save guard for reducing neonatal mortality as well as childhood morbidity and mortality. Overall, the findings of the study have paved the way for policy makers to implement different interventions aimed at improving the accessibility and utilization of all the components of antenatal care services among every single woman.

## 5. Conclusion

The study has revealed that utilization of antenatal care services among women aged 15–49 years is still insufficient in India. A sizable proportion of women did not fulfill the WHO-recommended criterion of obtaining ANC services. It is found that women’s decision-making autonomy regarding seeking health care services, physical movements such as friend or family visits, household purchasing, and spending money has a positive impact on the utilization of antenatal care services. Furthermore, women aged 25–34 years, who completed higher education, accessed to mass media, belonged to the richest household wealth quintile, and from southern, western, and eastern regions were more likely to seek ANC services. Therefore, it is recommended that appropriate measures should be adopted to eliminate gender bias and promote women’s autonomy along with extension of education, raising awareness about the importance of maternal health care services, and also elevate public health infrastructure so that everyone can access quality maternal health care services, that will eventually help in improving maternal health as well as societal health.

## List of abbreviations

ANC: Antenatal care
WHO: World health organization
NFHS National: Family Health Survey
COR: Crude odds ratio
AOR: Adjusted odds ratio
CI: Confidence interval
